# ADAM33 gene silencing by promoter hypermethylation as a molecular marker in breast invasive lobular carcinoma

**DOI:** 10.1186/1471-2407-9-80

**Published:** 2009-03-06

**Authors:** Gerusa G Seniski, Anamaria A Camargo, Daniela F Ierardi, Edneia AS Ramos, Mariana Grochoski, Enilze SF Ribeiro, Iglenir J Cavalli, Fabio O Pedrosa, Emanuel M de Souza, Silvio M Zanata, Fabrício F Costa, Giseli Klassen

**Affiliations:** 1 Department of Basic Pathology, Federal University of Parana, PR, Brazil; 2 Laboratory of Molecular Biology and Genomics, Institute Ludwig for Cancer Research, SP, Brazil; 3 Pediatric Oncology Institute/GRAACC, SP, Brazil; 4 Department of Genetics, Federal University of Parana, PR, Brazil; 5 Department of Biochemistry and Molecular Biology, Federal University of Parana, PR, Brazil; 6 Cancer Biology and Epigenomics Program, Children's Memorial Research Center and Northwestern University's Feinberg School of Medicine, Chicago, IL, USA

## Abstract

**Background:**

ADAM33 protein is a member of the family of transmembrane glycoproteins composed of multidomains. ADAM family members have different activities, such as proteolysis and adhesion, making them good candidates to mediate the extracellular matrix remodelling and changes in cellular adhesion that characterise certain pathologies and cancer development. It was reported that one family member, *ADAM23*, is down-regulated by promoter hypermethylation. This seems to correlate with tumour progression and metastasis in breast cancer. In this study, we explored the involvement of ADAM33, another ADAM family member, in breast cancer.

**Methods:**

First, we analysed *ADAM33 *expression in breast tumour cell lines by RT-PCR and western blotting. We also used 5-aza-2'-deoxycytidine (5azadCR) treatment and DNA bisulphite sequencing to study the promoter methylation of ADAM33 in breast tumour cell lines. We evaluated ADAM33 methylation in primary tumour samples by methylation specific PCR (MSP). Finally, *ADAM33 *promoter hypermethylation was correlated with clinicopathological data using the chi-square test and Fisher's exact test.

**Results:**

The expression analysis of *ADAM33 *in breast tumour cell lines by RT-PCR revealed gene silencing in 65% of tumour cell lines. The corresponding lack of ADAM33 protein was confirmed by western blotting. We also used 5-aza-2'-deoxycytidine (5-aza-dCR) demethylation and bisulphite sequencing methodologies to confirm that gene silencing is due to *ADAM33 *promoter hypermethylation. Using MSP, we detected *ADAM33 *promoter hypermethylation in 40% of primary breast tumour samples. The correlation between methylation pattern and patient's clinicopathological data was not significantly associated with histological grade; tumour stage (TNM); tumour size; ER, PR or ERBB2 status; lymph node status; metastasis or recurrence. Methylation frequency in invasive lobular carcinoma (ILC) was 76.2% compared with 25.5% in invasive ductal carcinoma (IDC), and this difference was statistically significant (p = 0.0002).

**Conclusion:**

*ADAM33 *gene silencing may be related to the discohesive histological appearance of ILCs. We suggest that *ADAM33 *promoter methylation may be a useful molecular marker for differentiating ILC and IDC.

## Background

ADAM33 protein is a member of the family of transmembrane glycoproteins composed of multidomains [1]. *ADAM33 *was originally identified as an asthma-susceptibility gene. Several single-nucleotide polymorphisms have been associated with asthma and bronchial hyperresponsiveness [2]. ADAM33 protein contains an active site sequence with a zinc-binding motif. It contains a glutamic acid in the catalytic domain, and therefore it is an active metalloproteinase [1,3]. Despite the fact that the physiological function of ADAM33 remains unknown, it is capable of cleaving stem cell factor (SCF) *in vitro *[3]. SCF and its receptor KIT which is known to be an oncoprotein are involved in several cancer types [3]. The disintegrin domain in ADAM33 can mediate integrin α9β1-dependent cell adhesion [4] and can inhibit α5β1 integrin-mediated cell migration [5]. The *ADAM33 *gene has been mapped to human chromosome 20p13, and it consists of 22 exons [1]. The gene has CpG islands within its promoter region. It was recently reported that *ADAM33 *is silenced by methylation in airway epithelial cells. It shows hypomethylation in mesenchymal cells, suggesting that methylation controls expression in a cell type-specific manner [6].

Recently, aberrant epigenetic gene silencing in cancer has been reported by different groups. These findings show the implication of these mechanisms in cancer development [7]. *ADAM23*, one of the ADAM family members, has been studied previously [8]. That study implicated promoter hypermethylation in transcriptional silencing in breast tumours at a more advanced stage [8]. In breast cancer, a variety of critical genes have been shown to be silenced by methylation (e.g., *BRCA1*, *14-3-3*, *TIM3*, *ESR1*, *PGR *and *E-cadherin*) [9].

In the present study, we investigated whether the *ADAM33 *gene in breast tumours is regulated by epigenetic mechanisms such as DNA methylation.

## Methods

### Patient samples

Frozen samples of breast tissue (n = 72) used for methylation analysis were obtained from patients treated by primary surgery for breast cancer at the Nossa Senhora das Graças Hospital, Curitiba, Parana, Brazil, with institutional approval. All patients gave informed consent for the study to retain and analyse their tissue for research purposes. The study included only female patients with primary invasive breast cancer. The age of patients ranged from 27 years to 84 years (mean 59.1 ± 13.1 years). In most cases, the histologic type was either infiltrative ductal carcinoma (IDC, n = 51 [70.8%]) or infiltrative lobular carcinoma (ILC, n = 21 [29.2%]). Histologic grade was determined according to the modified Bloom-Richardson criteria as Grade 1 in 27.2% of tumours, Grade 2 in 47.1% of tumours, and Grade 3 in 25.7% of tumours. TNM staging was done according to the official classification [10]. Immunohistochemical stains were evaluated and scored by two pathologists who were responsible for the clinicopathological data. The cut-off value for estrogen receptor (ER) and progesterone receptor (PR) status was 5% of cells. The ERBB2 data were obtained with a HercepTestTM (DAKO A/S, Grostrup, Denmark). When a result of +2 positive was obtained, an *in situ *fluorescent hybridization (FISH) assay was performed to confirm the result. Other clinicopathological data (tumour size, lymph node status, local recurrence and metastasis) are summarised in Table 1.

**Table 1:** Clinicopathological parameters compared to *ADAM33 *promoter methylation analyzed by Chi-square Test or Fisher's Exact Test.

Variate	*n*^a ^(%)	*ADAM33 *Methylation (%)		p-value
		Positive	Negative	
**Tumor Stage**				
0/I	14 (20.0)	4 (28.6)	10 (71.4)	0.641
II	33 (47.1)	14 (42.4)	19 (57.6)	
III/IV	23 (32.9)	8 (34.8)	15 (65.2)	
**Tumor Size**^b^				
pT1/pTis	20 (27.4)	6 (30.0)	14 (70.0)	0.174
pT2	38 (52.1)	14 (36.8)	24 (63.2)	
pT3/pT4	15 (20.5)	9 (60.0)	6 (40.0)	
**Histological Grade (SBR)**				
I	19 (27.1)	8 (42.1)	11 (57.9)	0.799
II	33 (47.1)	14 (42.4)	19 (57.6)	
III	18 (25.7)	6 (33.3)	12 (66.7)	
**Lymph Node Status**				
Negative	34 (47.9)	13 (38.2)	21 (61.8)	0.965
Positive	37 (52.1)	15 (40.5)	22 (59.5)	
**Estrogen Receptor Status (ER)**				
Negative	12 (17.1)	4 (33.3)	8 (66.7)	0.751
Positive	58 (82.9)	24 (41.4)	34 (58.6)	
**ERBB2 Status**				
Negative	43 (66.2)	18 (41.9)	25 (58.1)	0.378
Positive	22 (33.8)	6 (27.3)	16 (72.7)	
**Progesterone Receptor Status (PR)**				
Negative	15 (24.6)	4 (26.7)	11 (73.3)	0.678
Positive	46 (75.4)	17 (36.9)	29 (63.1)	
**Metastasis Status**				
Negative	55 (79.7)	23 (41.8)	32 (58.2)	0.548
Positive	14 (20.3)	4 (28.6)	10 (71.4)	
**Recurrence Status**				
Negative	63 (91.3)	23 (36.5)	40 (63.5)	0.201
Positive	6 (8.7)	4 (66.7)	2 (33.3)	
**Histological Tumor Type**				
Invasive Ductal Carcinoma	51 (70.8)	13 (25.5)	38 (74.5)	0.0002
Invasive Lobular Carcinoma	21 (29.2)	16 (76.2)	5 (23.8)	

^a^Only female patients with primary invasive breast cancer were included. ^b^According to TNM classification [10].

### Cell lines

Total RNA was kindly provided by Dr Michael O'Hare from the Ludwig Institute for Cancer Research (University College, England) from the following breast tumour cell lines: MDA-MB-134, MDA-MB-415, MDA-MB-175, MDA-MB436, MDA-MB-435, MDA-MB-468, MDA-MB456, BT-20, ZR-75-30, ZR-75-1, CAMA-1, GI101, 734B, CAL51, MCF7, SK-B-7; SK-BR-5, SK-BR-3, PMC42 and DU4475. The following breast cell lines, all obtained from the Ludwig Institute for Cancer Research (São Paulo, Brazil) were cultured in this study: HB4a (normal epithelial immortalised) [11], MDA-MB-231, MDA-MB-436, MDA-MB-435, MCF7 and PMC42. The cell lines were cultured in RPMI 1640 medium (GIBCO/Invitrogen Life Technologies, USA) containing 10% foetal bovine serum (complemented with 0.2 mM glutamine, 40 μg/mL garamycin and 10 μg/mL insulin) at 37°C in a humidified incubator with 5% CO_2_.

### DNA/RNA isolation of breast cell lines and breast cancer cells

Frozen tissue samples were dissolved in lysis buffer for subsequent DNA isolation using the phenol/chloroform protocol. They were then subjected to sodium bisulphite treatment using the EpiTect^® ^Bisulfite Kit (Qiagen). For total RNA isolation, the TRIzol Reagent (Life Technologies, USA) was used according to the protocol supplied by the manufacturer.

### *ADAM33 *expression pattern

ADAM33 mRNA expression analysis was performed by applying RT-PCR to an RNA panel of 20 breast tumour cell lines and normal breast. HB4a was included as a normal cell line control. Reverse transcription reactions were performed using 500 ng of DNA-free RNA, an oligo(dT)_12–18 _primer and Superscript II Reverse Transcriptase (Gibco, BRL). PCR was performed using *ADAM33*-specific primers. The sense primer was 5' CAT GAC ACC TTC ATG CTG, and the anti-sense primer was 5' ATC TTG GCA TCT GGA CTT G. The PCR was performed in a volume of 20 μl containing 1× PCR buffer (Invitrogen), 1.5 mM MgCl_2 _(Invitrogen), 200 μM dNTPs (Gibco, BRL), 0.30 μM of each primer and 1 U of *Taq *Platinum (Invitrogen). The PCR conditions were as follows: 95°C for 12 min, 94°C for 45 s, 60°C for 45 s, 72°C for 1 min and a final extension of 72°C for 5 min. Primers for *GAPDH *were as follows: sense, 5' CTG CAC CAC CAA CTG CTT A; anti-sense, 5' CAT GAC GGC AGG TCA GGT C. PCR conditions were as follows: 95°C for 12 min, 94°C for 45 s, 63°C for 45 s, 72°C for 1 min and a final extension of 72°C for 5 min. PCR products were resolved on 1% agarose gels and visualised by ethidium bromide staining. Hybridisations were carried out as previously described [12] using *ADAM33 *or *GAPDH *cDNA probes fragments corresponding to nucleotides 2191–2515 and 486–778, respectively. They were amplified using *ADAM33 *RT-PCR and *GAPDH *RT-PCR primers, purified from agarose gels and labelled with ^32^P.

### Western Blotting

Western blotting to detect ADAM33 protein was performed on the HB4a normal breast cell line (positive for *ADAM33 *mRNA expression) as well as on the MDA-MB-231 and MDA-MB-435 (both negative for *ADAM33 *mRNA expression) tumour cell lines. Protein lysates were obtained from approximately 1.2 × 10^7 ^cells in lysis buffer (50 mM Tris-HCl, pH 7.4, 0.5% Triton X-100 and 0.2% sodium deoxycholate) containing protease inhibitors (Complete, Roche). Protein samples (100 μg) were resolved by one-dimensional 7.5% SDS-PAGE. Molecular mass was estimated by comparison with Rainbow Molecular Weight Markers (RPN 756, Amersham). Proteins were transferred to PVDF membranes by electroblotting. A commercial polyclonal antibody (Sigma) against the catalytic domain of ADAM33 was used for immunodetection. Horseradish peroxidase-conjugated anti-rabbit secondary antibody was used to detect the binding of primary antibody. HSP70 was detected on the blots as a control of protein integrity.

### 5-aza-2'deoxycytidine (5-aza-dCR) treatment

The cell lines MDA-MB-231, MDA-MB-435, and MDA-MB-436 were analysed using this technique. Cells (10^6^) were incubated with 1 μM of 5-aza-dCR (Sigma Aldrich, Deisenhein, Germany) or left untreated. Every day the medium was changed and no significant cell death was observed. After seven days of treatment, a reduction in cell number was observed, and total RNA was extracted. The expression of *ADAM33 *in breast tumour cells was analysed by RT-PCR using the housekeeping gene *GAPDH *as an internal control. PCR products were visualised on a silver-stained 8% polyacrylamide gel. MM 10 bp (Invitrogen) were used as molecular weight markers.

### *ADAM33 *CpG Island methylation analysis

Identification of the *ADAM33 *CpG island was accomplished using the human genome sequence corresponding to the promoter region of the transcription start site (TSS) of the *ADAM33 *gene. We identified the RefSeq number based on the GenBank accession and submitted the gene sequence to the Blat Search Genome at the UCSC Genome bioinformatics website http://genome.ucsc.edu. We selected 2000 bp of sequence extending from the 5'-upstream region to 1000 bp downstream region of the TSS. The sequence was submitted for analysis to the CpGPLOT program from the European Bioinformatics Institute website http://www.ebi.ac.uk/emboss/cpgplot. Typical CpG islands were defined as ≥ 200 bp of sequence that had a C+G content of ≥ 50% and a value of >0.6 for the ratio (CpG observed)/(CpG expected) [13]. The selected region of -472 to +389 was amplified from bisulphite-treated DNA samples using a nested-PCR amplification protocol. The first set of primers included a sense primer 5' AGG GAG TTA TGT TTT TTG TTT TGT TAG and an anti-sense primer 5' ATT ACC TAA ACC TTC CTA TCC TTA. PCR products were used as templates for the nested PCR. The second set of primers included a sense primer 5' GGG TTA GTT TAA GTA TAT TTG AG and an anti-sense primer 5' ACA CCC AAT ACA AAT AAA TAA CC. The PCR conditions were as follows: one round of 95°C for 12 min, 94°C for 3 min, 48°C for 3 min, 72°C for 2 min; five cycles of 94°C 3 min, 50°C for 3 min, 72°C for 2 min; and 35 cycles of 94°C for 1 min, 52°C for 1 min, and 72°C. Different annealing temperatures (55°C, 57°C and 59°C) were used for the nested reaction. Amplified products were purified using the Qiaquick Gel Extraction Kit (Qiagen) and cloned into a pCR2.1 cloning vector (Invitrogen). Eight clones were sequenced for each cell line using the vector's universal and/or reverse primers. DNA sequencing reactions were performed using Big Dye Terminator technology (Applied Biosystems) on an ABI 377 sequencer (Applied Biosystems) according to the manufacturer's instructions. The 100% of methylation was obtained if cytosine in the CpG dinucleotides was present in the eight sequenced clones. The methylation percentage for each tumour cell lines (global methylation pattern) was calculated by dividing the number of methylated CpG dinucleotides by the total number of CpGs analysed.

### *ADAM33 *Methylation-Specific PCR

Methylation-specific PCR (MSP) was performed as previously described [14]. MSP primers for the methylated condition (M) included a sense primer 5'GTT TGA GGT TGT ATC GGG TA and an anti-sense primer 5'ACT CGC AAC TCC GAC TCC G. For the unmethylated condition (U), a sense primer 5' GTT TGA GGT TGT ATT GGG TA and an anti-sense primer 5' ACT CAC AAC TCC AAC TCC A were used. The PCR protocol was one round of 95°C for 10 min; 35 cycles of 94°C for 45 s and either 66°C for methylated condition (M) or 64°C for unmethylated condition (U) for 15 s, followed by 72°C for 45 s; and a final extension of 72°C for 5 min. PCR products were run on 8% polyacrylamide gels and silver stained.

### Statistical analysis of clinicopathological patient data

Statistical analyses were carried out using SPSS (version 12.0; SPSS, Chicago, IL). Differences were considered statistically significant when P-values were below 0.05.

## Results

### *ADAM33 *expression pattern

**Figure 1:**
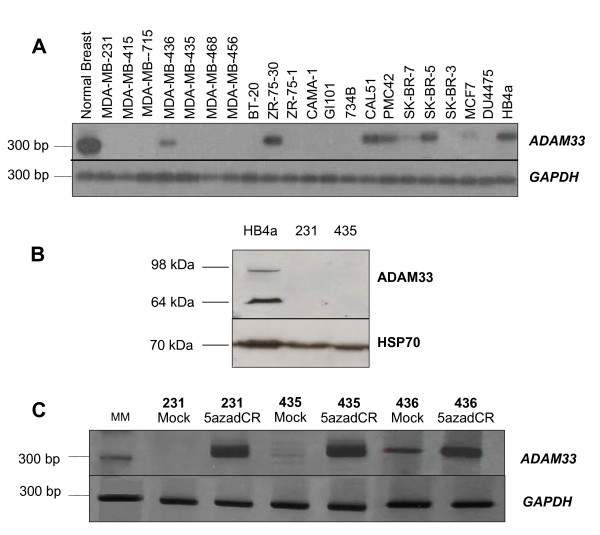
***ADAM33 *mRNA and protein expression**. **(A) **The *ADAM33 *RNA panel of breast cell lines, normal breast and HB4a normal cells. The expected products corresponding to *ADAM33 *(328 bp) and *GAPDH *(290 bp) are showed. **(B) **ADAM33 western blotting. The ADAM33 bands from mature and cleaved forms (98 and 64 kDa) ADAM33 signal. HSP70 was used as control. **(C) ***ADAM33 *re-expression after 5-azadCR treatment. MM (molecular marker 100 bp). The expected products were to *ADAM33 *(328 bp) and *GAPDH *(290 bp).

Using RT-PCR followed by Southern blotting hybridisation, we evaluated *ADAM33 *mRNA expression in a panel of 20 breast tumour cell lines, normal breast tissue and a normal breast cell line (HB4a). *ADAM33 *expression was detected at high levels in normal tissue and in the normal breast cell line, but it was not detected in 13 (65%) of the 20 breast tumour cell lines (Figure 1A). Western blot analysis using a specific anti-ADAM33 antibody showed that the protein was present in the positive control (cell line HB4a) that expressed *ADAM33 *mRNA, but not in the MDA-MB231 or MDA-MB435 cell lines, which also lacked *ADAM33 *mRNA (Figure 1B). These results indicate that *ADAM33 *is down-regulated at both the transcriptional and translational levels in breast tumour cell lines. Two bands reacting with ADAM33 antibody were detected at the molecular weights of 98 and 64 kDa, which probably correspond, respectively, to the mature form of ADAM33, which has been processed and glycosylated, and to the truncated form, which has been cleaved near the transmembrane region [15-17].

### Mechanism of *ADAM33 *silencing by DNA methylation

To investigate the mechanism of *ADAM33 *transcriptional silencing, we treated MDA-MB-435, MDA-MB-436 and MDA-MB-231 breast tumour cell lines with the demethylating agent 5'-aza-2'deoxycytidine (5-azad-CR). The expression of *ADAM33 *was restored upon treatment in all cell lines, as detected by RT-PCR (Figure 1C).

### *ADAM33 *CpG island methylation analyses by sodium bisulphite sequencing

**Figure 2:**
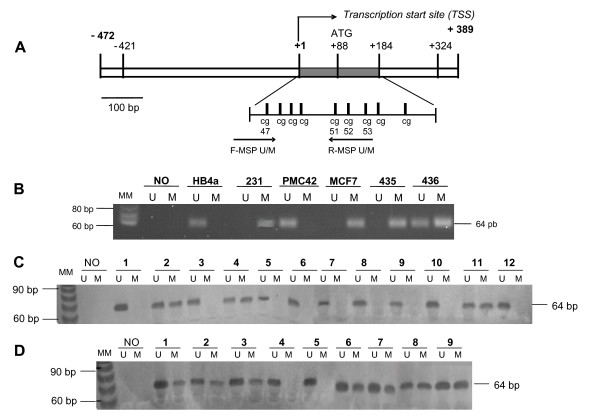
***ADAM33 *Methylation-Specific PCR in cell lines and in primary breast tumours. (A) **Schematic representation of the sequenced region of the *ADAM33 *gene. Positions in base pairs were calculated from the transcription start site (+1). The region comprises a CpG island (-421 and +324). The first exon is indicated at nucleotides +1 and +184 as a grey bar. The zoom shows the dinucleotide CpG in the chosen region after sequencing with designed MSP primers. The *arrows *represent MSP primers. The primers in F unmethylated (U) or methylated (M) contain the CpG dinucleotide 47 and the primers in R (U or M) contain the CpG dinucleotides 51, 52 and 53. **(B) **MSP of *ADAM33 *in breast carcinoma cell lines. The marker ladder 10 bp (Invitrogen) was used (MM). In the negative control (NO), water was used instead of template. The presence of a visible PCR product (64 bp) in the lanes marked *U *indicates the presence of the unmethylated *ADAM33 *gene. The presence of a product in the lanes marked *M *indicates the presence of methylated *ADAM33 *gene. **(C) **MSP of *ADAM33 *in primary breast invasive ductal carcinomas (64 bp). MSP results from 12 representative patients are shown. **(D) **MSP of *ADAM33 *in primary breast invasive lobular carcinomas (64 bp). MSP results from nine representative patients are shown.

**Figure 3:**
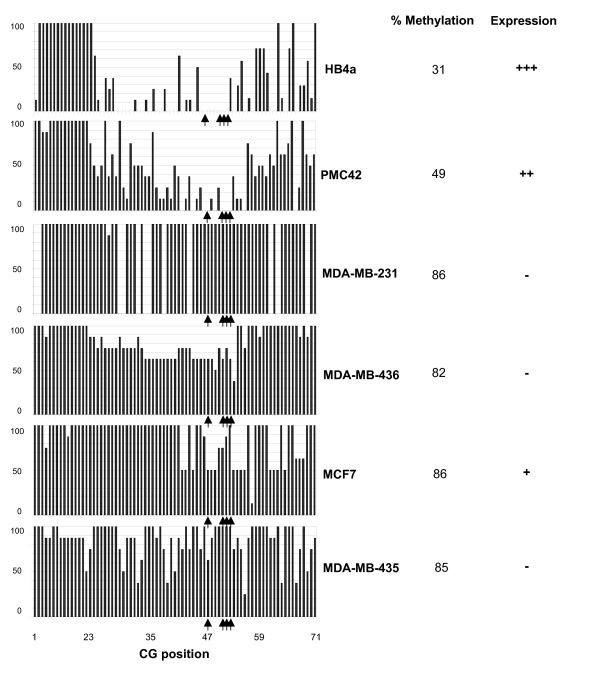
**Cytosine methylation of the *ADAM33 *CpG island from a normal breast cell line and in breast tumour cell lines**. 100% methylation means that the cytosine in that position is methylated in all eight clones sequenced. The CpGs position are indicate below. The arrows indicate the CpGs used for MSP primers. The global methylation and the expression status are indicated on the right.

Two putative islands were found in the *ADAM33 *promoter region. One of the CpG islands is located between -1012 and -748. This island has 264 bp, proximal to the cut-off for a CpG island [13]. For this reason, we decided to study the region from -421 to +324, which contains part of the first exon. This region possesses promoter activity, as previously described by others [6], as well as more than 40 transcription factor-binding sites predicted by the TFSEARCH software (data not shown). Sodium bisulphite sequencing was carried out on a region containing 77 CpG dinucleotides (-472 to +389), 71 of which were located within one of the CpG islands (-421 to +324) of the TSS (Figure 2A). We analysed the methylation pattern of eight independent alleles (eight clones) from normal HB4a, as well as the breast tumour cell lines PMC42, MDA-MB-231, MDA-MB-435, MDA-MB-436 and MCF7. Methylation of more than 80% of the CpG dinucleotides in this region was detected in the breast tumour cell lines analysed except PMC42 (Figure 3). All cell lines that exhibited hypermethylation showed down-regulation of *ADAM33 *expression (Figure 1A). We observed that there are some dinucleotides that are likely to be more important than others in transcriptional regulation. Comparing Figure 1A with Figure 3, it is clear that *ADAM33 *transcripts from cell lines with a lower methylation density were expressed at higher levels than in cells with a higher methylation density. However, MCF7 has its *ADAM33 *promoter region highly methylated (methylation percentage = 86.1%), and transcription occurs to a smaller extent (Figure 1A). These results indicate that there are crucial CpGs that must be methylated in order to completely silence gene transcription. These results also indicate that the methylation density is critical to the down-regulation of expression. In other words, hypermethylation may not be the only factor of critical importance to transcription. These dinucleotides may be important for the binding of the transcription machinery, mainly because they are located near the TSS. The methylation seems to initiate mostly at dinucleotides 1–3 and 74–77 and to spread towards the promoter region. These results may therefore support the spreading model proposed by Turker [18]. Moreover, methyl-binding proteins physically associate with methylated CpG and attract repressive complexes such as histone deacetylases (HDAC) [19]. Different histone tail modifications may exist. It is possible that the level of gene expression for a given allele can vary from extremely high to undetectable. Alternatively, repression by the methyl-CpG-binding proteins can occur via mechanisms that do not involve histones [19]. Another possibility is that the biallelic inactivation of tumour suppressor genes involves DNA methylation, deletion or point mutation. Furthermore, the sequence of inactivating events can occur in any order [20].

### MSP analysis in primary breast tumours

The dinucleotides 47, 51, 52 and 53 lie within a region that is differentially methylated in order to regulate expression of *ADAM33 *(Figure 3). This region was chosen for the design of methylation-specific PCR (MSP) primers (Figure 2A). In the MSP reactions we tested the breast cancer cell lines MDA-MB-231, MCF7, PMC42, MDA-MB-435, and MDA-MB-436 as well as the normal cell line HB4a (Figure 2B). The MSP results corroborated the bisulphite sequencing and RT-PCR data for the cell lines. This assay was subsequently used to analyse breast primary tumour samples. Representative tumour MSP for IDC or ILC results are shown (Figure 2C and 2D respectively). Methylation was frequently observed in ILC (Figure 2D). The presence of contaminating normal tissue or infiltrating lymphocytes explains the unmethylated DNA in all tumour samples.

**Figure 4:**
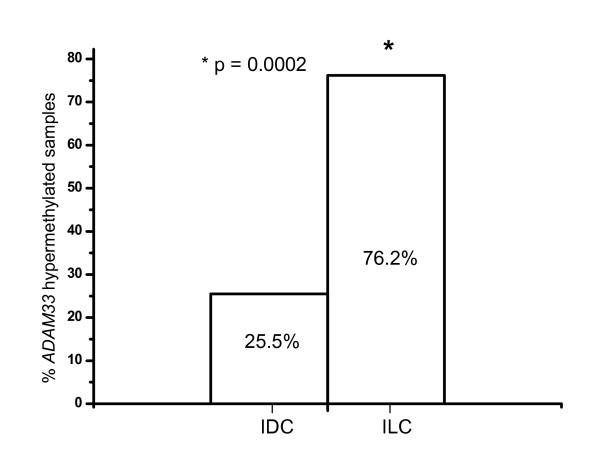
***ADAM33 *methylation analyses of invasive ductal carcinomas (IDC) and invasive lobular carcinomas (ILC)**. The frequency of methylation was 76.2% for ILC samples and 25.5% for IDC samples. * Indicates statistical significance (p = 0.0002)

Descriptive chi-square and Fisher's exact tests were performed in order to correlate *ADAM33 *promoter methylation with clinical and pathological features (Table 1). *ADAM33 *promoter hypermethylation was observed in 29 of 72 (40.3%) breast cancer specimens. In a separate analysis of the histological tumour types, the difference in methylation frequency for IDC and ILC was statistically significant (25.5% in IDCs and 76.2% in ILCs; p = 0.0002) (Table 1, Figure 4).

Abnormal methylation of the *ADAM33 *promoter region in breast carcinomas was not associated with any significant difference in tumour stage (p = 0.641), histological differentiation grade (SBR; p = 0.799), lymph node status (p = 0.965), hormone receptor status (ER, p = 0.751; PR, p = 0.678), ERBB2 status (p = 0.378) or metastasis status (p = 0.548). The methylation frequency for all of these variables was approximately 30–40% (Table 1). Although no statistically significant correlation was found between these variables and the degree of methylation, some interesting features may justify future investigation. For example, methylation frequency was higher in tumours of size T3 and T4 than in tumours of smaller size. The methylation frequencies were similar for tumours of size T1 (30.0%) and T2 (36.8%), whereas frequencies of up to 60% were observed in tumours of size T3 and T4. However, these differences were not statistically significant (p = 0.174). Another potentially important observation is that in samples showing local tumour recurrence, the *ADAM33 *promoter methylation frequency was 66.7%, compared to 36.5% for non-recurrent samples (p = 0.201).

## Discussion

Many studies have shown that ADAM family members function in fundamental processes such as cell adhesion, cell fusion, cell migration, membrane protein shedding and proteolysis. Moreover, the shedding activity of cytokines and growth factors seems to be related to cellular migration and to the control of several signalling pathways activated in cancer. For this reason, it is not surprising that deregulated expression of ADAM family members has been reported in human tumours [21]. In agreement with this observation, our results show that the *ADAM33 *gene is down-regulated. This probably occurs by selective DNA hypermethylation in many breast carcinomas, especially those of the invasive lobular histological type (ILC).

ILC is less common than IDC, accounting for 10–15% of breast cancers [22]. This tumour type may appear as a palpable IDC-like mass. More frequently, however, its cells grow in single linear rows around ducts and lobules. This so-called "Indian file" pattern of infiltration causes a modest disruption of the underlying anatomic structures and generates little surrounding desmoplastic stromal reaction. This limited reaction makes diagnosis difficult even by mammography and ultrasound [22]. The most obvious characteristic of this histological tumour type is the loss of E-cadherin expression, which is the major adhesion protein in breast epithelium. Its absence explains the histological morphology of ILC. Loss of adhesion may also explain why ILCs tend to metastasise to more remote locations, such as gastrointestinal and female reproductive systems, whereas IDCs metastasise preferentially to lung, liver and bones [23]. It is important to note, however, that not all lobular carcinomas show a complete loss of E-cadherin expression. In some cases, ILCs have low levels of E-cadherin expression, implying that alterations in the expression of other cell-to-cell adhesion proteins likely occur [24]. ADAM33 may be one such protein, since a protein of the same family (ADAM23) is involved in cell adhesion. Moreover, it has recently been shown that ADAM33 is capable of interacting with integrins, suggesting its involvement in cell adhesion [4].

More studies about ADAM33 protein function in cancer are necessary to understand its tumour-suppressor role. In addition, we speculate that ADAM33 is involved with the *KIT *oncogene pathway in cancer, given that the ADAM33 catalytic domain is capable of cleaving SCF (Kit ligand) *in vitro *[25]. We suggest the possible usefulness of both *ADAM33 *promoter methylation and the lack of the protein as molecular diagnostics to differentiate IDC and ILC. Such a differential marker may be of great value in the clinic, since IDCs and ILCs are similar in many respects and their histological features occasionally overlap, primarily in cases of mixed carcinomas [26]. To date, E-cadherin is the only well-established immunohistochemical marker for the differentiation of ductal and lobular breast carcinomas, and few studies describe useful molecular markers for this or other purposes [26,27]. Lobular tumour metastases in the gastrointestinal system do not form glands or tubular structures but instead infiltrate as small nests and strands. These are usually of the "signet-ring" type, making histopathological diagnosis difficult. Moreover, ILC usually has a low mitotic rate and a uniform appearance. It tends to infiltrate lymph nodes in a single-cell pattern, which makes distinguishing between lobular carcinoma cells and lymphoid cells extremely challenging [22]. Effectively distinguishing between ILC and IDC may be important for determining the most appropriate treatment, since change the two types of tumour respond differently to therapy. In fact, ILCs are often resistant to neoadjuvant therapy.

## Conclusion

In summary, our findings suggest that *ADAM33 *is a novel tumour suppressor gene that may be useful as a molecular marker for invasive lobular carcinoma of the breast. To our knowledge, this is the first study to associate *ADAM33 *with breast cancer and the only data that associate *ADAM33 *with a particular cancer type. Further studies are needed to determine the immunohistochemical the ADAM33 protein profile. Further studies are also needed to determine the prognostic and predictive significance of *ADAM33 *methylation and silencing in invasive lobular carcinoma.

## Competing interests

The authors declare that they have no competing interests.

## Authors' contributions

GGS, carried out the experimental data acquisition, performed data analyses and interpretation and drafted the manuscript. AAC designed the study and critically revised the manuscript. EASR processed clinical samples for MSP analyses. MG processed cell cultures and DNA extractions. ESFR and IJC provided patient material and clinicopathological data and critically revised the manuscript. FOP critically revised the manuscript and gave equipment support. DFI participated in the RT-PCR and hybridisation analysis. EMS critically revised the manuscript and suggested experiments. SMZ analysis and critically revised the manuscript. FFC helped in the experimental design and critically revised the manuscript. GK designed and coordinated the study, supplied administrative support and critically revised the manuscript. All authors read and approved the final manuscript.

## Pre-publication history

The pre-publication history for this paper can be accessed here:

http://www.biomedcentral.com/1471-2407/9/80/prepub
